# Pharmacokinetic Interaction of Kratom and Cannabidiol in Male Rats

**DOI:** 10.3390/pharmaceutics16030318

**Published:** 2024-02-24

**Authors:** Erin C. Berthold, Shyam H. Kamble, Siva Rama Raju Kanumuri, Michelle A. Kuntz, Alexandria S. Senetra, Yi-Hua Chiang, Sushobhan Mukhopadhyay, Christopher R. McCurdy, Abhisheak Sharma

**Affiliations:** 1 Department of Pharmaceutics, College of Pharmacy, University of Florida, Gainesville, FL 32610, USA; erincberthold@plantedinscience.com (E.C.B.); kanumuris@ufl.edu (S.R.R.K.); senetraa@ufl.edu (A.S.S.); chiangyihua@ufl.edu (Y.-H.C.); cmccurdy@cop.ufl.edu (C.R.M.); 2 Translational Drug Development Core, University of Florida Clinical and Translational Science Institute, Gainesville, FL 32610, USA; 3 Department of Medicinal Chemistry, College of Pharmacy, University of Florida, Gainesville, FL 32610, USA; smukhopadhyay@ufl.edu

**Keywords:** kratom, cannabidiol, pharmacokinetic interactions, mitragynine

## Abstract

Kratom and cannabidiol products are used to self-treat a variety of conditions, including anxiety and pain, and to elevate mood. Research into the individual pharmacokinetic properties of commercially available kratom and cannabidiol products has been performed, but there are no studies on coadministration of these products. Surveys of individuals with kratom use history indicate that cannabidiol use is one of the strongest predictors of both lifetime and past month kratom use. The purpose of this study was to determine if there are changes in pharmacokinetic properties when commercially available kratom and cannabidiol products are administered concomitantly. It was found that with concomitant administration of cannabidiol, there was a 2.8-fold increase in the exposure of the most abundant kratom alkaloid, mitragynine, and increases in the exposure of other minor alkaloids. The results of this work suggest that with cannabidiol coadministration, the effects of kratom may be both delayed and increased due to a delay in time to reach maximum plasma concentration and higher systemic exposure of the psychoactive alkaloids found in kratom.

## 1. Introduction

**Figure 1:**
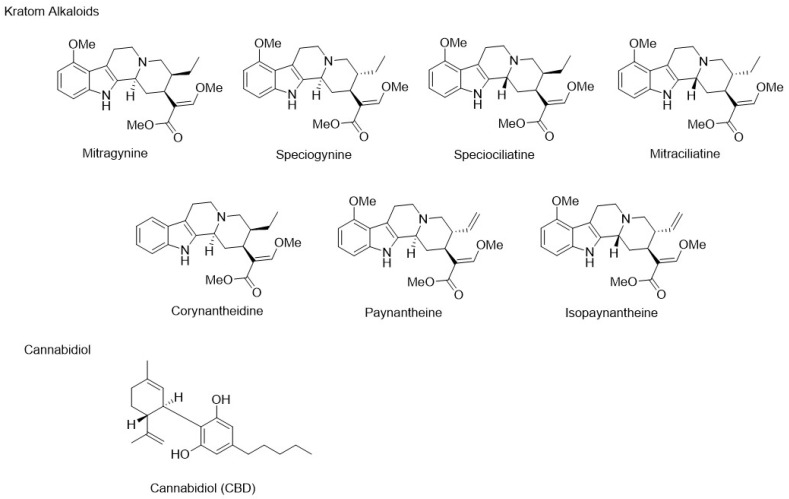
Chemical structures of select kratom alkaloids and cannabidiol.

Kratom and cannabidiol products are used individually to self-treat a variety of conditions, including anxiety, pain, and insomnia [1,2]. Cannabidiol (Figure 1) is a cannabinoid from the plant *Cannabis sativa* L. that produces no intoxication, and its mechanism of action is unclear [1,3,4]. Some of the known molecular targets of cannabidiol, either as an agonist, antagonist, or inhibitor, include cannabinoid receptors, G-protein-coupled receptors, serotonergic receptors, dopamine receptors, and a variety of transient receptor potential cation channels and vanilloid receptors [5]. Kratom products are derived from *Mitragyna speciosa* Korth., a Southeast Asian tree, and contain multiple psychoactive compounds [6]. Approximately 14% of Americans use cannabidiol, while there are an estimated 2–15 million kratom users in the United States [1,2,7]. 

Commercially available kratom products contain multiple bioactive alkaloids, with the most abundant being mitragynine (Figure 1). Mitragynine displays mixed pharmacological effects in vivo with activity at opioid, adrenergic, serotonergic, and dopamine receptor subtypes [6]. Major and minor alkaloids found in kratom include speciociliatine, speciogynine, paynantheine, isopaynantheine, mitraciliatine, and corynantheidine (Figure 1). All of the aforementioned alkaloids, except corynantheidine, showed measurable systemic exposure in humans after a 2 g dose of kratom leaf brewed into a tea [8]. These minor alkaloids all have their own unique and complex receptor-binding characteristics that are not well understood. Some of the central nervous system (CNS) receptor subtypes that are known targets of kratom alkaloids include µ-, δ-, and κ- opioid receptors, adrenergic receptors alpha_1A_ and alpha_2A_, serotonergic receptors 5-HT_1A_ and 5-HT_2A_, and dopamine D_1_ and D_2_ receptors [6]. 

In addition to the multitude of receptor subtypes purported to be activated by kratom alkaloids and cannabidiol, both kratom and cannabidiol have also been implicated as having potential for pharmacokinetic interactions both in vitro and in vivo [6,9,10,11,12,13,14,15,16,17,18,19,20,21,22,23,24,25]. Both cannabidiol and mitragynine are potential inhibitors of cytochrome P450 (CYP) 2D6, and kratom was found to increase the exposure of midazolam (CYP3A substrate) when taken concomitantly with a 2 g dose of leaf kratom tea in humans [10].

One study has investigated the pharmacodynamic interactions of coadministered mitragynine and cannabidiol in a mouse model of chemotherapy-induced peripheral neuropathy. The results showed an additive interaction to attenuate allodynia. There was also an increase in antinociception and a decrease in scheduled-controlled responding, indicating that the combination increases pain relief while also becoming sedating and/or intoxicating [26]. Surveys of kratom users have found that cannabidiol use is one of the strongest predictors of both past month kratom use (odds ratio 4.18; *p* < 0.001 in 289 kratom users) and lifetime kratom use (odds ratio 3.73; *p* < 0.001 in 202 kratom users) [27,28]. Another study of over 5000 kratom users found that 11.5% self-reported taking cannabidiol with kratom, second only to cannabis (12.5%) [29]. A recent review of kratom and cannabidiol for chronic pain demonstrates the need for more research, as these products are being consumed concomitantly by an appreciable number of Americans [30].

Due to the complicated pharmacology of kratom and the poorly understood mechanism of action of cannabidiol, it is essential that research into the interaction potential of these two natural products is performed, as kratom and cannabidiol are being utilized to self-treat a variety of conditions with no safety data and little to no controlled preclinical or clinical study on the interaction potential. Yet, these natural products are often considered “safer” because they are “plant-based”, so are used in conjunction with other substances. This is cause for concern, as they pose the same risks for interaction as any other pharmacologically active compounds [31,32,33]. To better understand the interaction of kratom and cannabidiol, this polysubstance combination was studied in rats. Both single- and multiple-dose pharmacokinetic studies were performed. The results of this study promote further understanding of the combined therapeutic potential, as well as highlighting potential risks of concomitant kratom and cannabidiol administration. 

## 2. Materials and Methods

### 2.1. Chemicals and Reagents

Cannabidiol isolate oil solution (33.3 mg/mL cannabidiol) was purchased from Organica Naturals (Sacramento, CA, USA). OPMS Gold liquid shot was procured through an online retailer (Choice Organics, Los Angeles, CA, USA). The metabolite of mitragynine, 7-hydroxymitragynine, was semi-synthesized from mitragynine, and individual kratom alkaloids, mitragynine, corynantheidine, speciogynine, speciociliatine, and paynantheine, were isolated and purified from a kratom alkaloid-rich extract as previously described (purity > 99%) [34]. Commercially available stock solutions (1 mg/mL) for cannabidiol and cannabidiol-d3 (internal standard for cannabinoids; IS-1) were obtained from Cerilliant (Round Rock, TX, USA). Verapamil hydrochloride was purchased from Sigma Aldrich (St. Louis, MO, USA) and used as an internal standard (IS-2) for the kratom alkaloids. LC-MS grade water, methanol, acetonitrile, and formic acid were sourced from Fisher Scientific (Fair Lawn, NJ, USA). Blank Sprague-Dawley rat plasma was purchased from Innovative Research Inc. (Novi, MI, USA). 

### 2.2. Animals and Dosing

Male Sprague-Dawley rats (weight 250 ± 25 g) were obtained from Envigo (Indianapolis, IN, USA) with indwelling jugular vein catheters. Animals were acclimated for ≥72 h in a temperature- and humidity-controlled vivarium on a 12 h light/dark cycle. All studies were approved by the University of Florida’s Institutional Animal Care and Use Committee (IACUC) and carried out in accordance with Association for Assessment and Accreditation of Laboratory Animal Care International (AAALAC) and National Institute of Health (NIH) guidelines. In the single-dose studies, rats were fasted for 10 h prior to dosing and an additional 2 h post-dose.

Kratom product control data for this study were from previous work done by Kamble et.al., where a 0.8 mL/kg dose of commercial kratom product (11.8 mg/mL mitragynine) was given to male Sprague-Dawley rats [35]. This equates to a human dose of 1.5 mg/kg mitragynine, which is one bottle of OPMS Gold solution (8.8 mL) consumed by a 70 kg individual. The same dose was used in this study for kratom (0.8 mL OPMS/kg body weight per day). For cannabidiol, a dose of 50 mg/kg per day was used for the control study. This is the human equivalent dose of 8 mg/kg, which falls within the range recommended for the Food and Drug Administration (FDA)-approved cannabidiol prescription drug, Epidiolex^®^ (5–20 mg/kg/day) [36]. 

For the cannabidiol alone study, 4 male rats were administered cannabidiol isolate product at a dose of 50 mg/kg. Blood samples were collected pre-dose, and 0.08, 0.17, 0.25, 0.50, 0.75, 1.0, 1.5, 2.0, 4.0, 8.0, 12, 18, and 24 h post-dose.

For the single-dose study, rats were pretreated with 50 mg/kg cannabidiol by oral gavage and, after 30 min, were dosed with 0.8 mL/kg OPMS Gold solution. A period of 30 min was observed between doses to reduce the likelihood of interactions between the formulations in the stomach. Rats were connected to an automatic blood collection system (BASi, West Lafayette, IN, USA), and blood samples were taken pre-kratom product dose and 0.08, 0.17, 0.25, 0.5, 1, 2, 3, 4, 6, 8, 12, 18, 24, 30, 36, 42, and 48 h after kratom product dose administration. For the multiple-dose study, rats were pretreated with 25 mg/kg cannabidiol and, after 30 min, were dosed with 0.4 mL/kg OPMS Gold solution every 12 h (0900 and 2100) for a period of 4 days. Blood samples were taken prior to the 0900 dose (24, 48, 72, 96 h). On the fifth dosing day, after the 0900 dose, rats were connected to an automatic blood collection system, and blood samples were collected at 96.17, 96.5, 97, 98, 100, 102, 106, 114, 120, 126, 132, 138, and 144 h after kratom product dose administration. Plasma was separated from blood by centrifugation at 850× *g* for 10 min at 4 °C. Samples were stored at −20 °C until analysis. 

### 2.3. Sample Preparation

Stock solutions were prepared separately for kratom alkaloids and cannabinoids but followed the same procedure. Each kratom alkaloid was weighed and dissolved in an appropriate organic solvent to obtain primary stock solutions of 1 mg/mL. Cannabinoid primary stock solutions were purchased at 1 mg/mL concentration. These primary stock solutions were used to make mix stocks of 10 µg/mL and 1 µg/mL of each compound. The mix stocks were then further diluted to provide calibration stocks (CS) of 50, 100, 250, 500, 1000, 1500, 2500, and 5000 ng/mL. Quality control (QC) stocks were prepared from a second set of mix stocks at concentrations of 50, 80, 2000, and 4000 ng/mL. 

Standards were prepared by adding 2.0 μL of CS or QC stock to 18 μL of blank rat plasma. This generated eight calibration standards (5–500 ng/mL) and four QC standards (5, 8, 200, and 400 ng/mL). After spiking, the standards were vortex-mixed for 5 min at 650 rpm. Analytes were precipitated with 80 μL methanol acidified with 0.05% formic acid containing 25 ng/mL IS-1 and IS-2 (Quenching Solution; QS). Samples were transferred to a 96-well filter plate and centrifuged at 850× *g* for 2 min at 4 °C. Study samples were prepared in the same manner by aliquoting 20 μL and adding 80 μL QS prior to vortex mixing and filtration. 

### 2.4. Sample Analysis

Samples were analyzed using previously validated ultraperformance liquid chromatography-tandem mass spectrometry (UPLC-MS/MS) methods for the quantification of cannabinoids and alkaloids in rat plasma [35,37]. Both methods used a Waters Acquity I-Class UPLC coupled to a Xevo TQ-S micro mass spectrometer (Waters, Milford, MA, USA). The source gas was nitrogen, and the collision gas was argon. The autosampler was maintained at 10 °C.

For the cannabinoid bioanalytical method, a 1.7 µm, 2.1 x 100 mm C18 BEH column was utilized with a pre-column of the same chemistry (Waters, Milford, MA, USA) held at 40 °C. The cannabinoid method had a mobile phase of water with 0.1% formic acid (A) and 50:50 methanol: acetonitrile [*v*/*v* (B)]. A gradient elution was used to separate cannabidiol at a flow rate of 0.35 mL/min, which started at 18% A and linearly decreased to 0% A over 4.5 min, followed by a steep return to initial conditions to re-equilibrate the column until 5.0 min. Cannabidiol was detected in positive electrospray ionization mode using multiple reaction monitoring. The instrument had a capillary voltage of 0.5 kV, a source temperature of 150 °C, a desolvation temperature of 450 °C, a desolvation gas flow of 800 L/h, and a cone gas flow of 60 L/h.

For the peak separations of kratom alkaloids required for quantification, a 1.7 µm, 3.1 mm × 100 mm C18 BEH column with a pre-column of the same chemistry (Waters, Milford, MA, USA) was utilized and held at 50 °C throughout the analysis. A gradient elution was used with mobile phase of aqueous ammonium acetate buffer [2.5 mM, pH 3.5 (A)] and acetonitrile (B). The gradient was held at 90% A for 1 min and decreased to 60% by 10 min. From 10 to 10.5 min, the percent of A was decreased sharply to 30% and was then set to initial conditions from 10.5 to 11 min to re-equilibrate the column. The alkaloids were detected in positive electrospray ionization mode using multiple reaction monitoring. The instrument had a capillary voltage of 0.5 kV, a source temperature of 150 °C, a desolvation temperature of 450 °C, a desolvation gas flow of 900 L/h, and a cone gas flow of 50 L/h. 

### 2.5. Data Analysis

The TargetLynx™ application of MassLynx™ was used for data processing and quantification of the UPLC-MS/MS data (Waters, Milford, MA, USA). Phoenix Version 6.4 (Certara, Princeton, NJ, USA) was used to perform a non-compartmental analysis of concentration–time data. GraphPad Prism Version 8 (GraphPad Software, San Diego, CA, USA) was used to generate all figures. 

The United States Food and Drug Administration provides guidance in determining the clinical significance of a drug–drug interaction. There are two approaches: one when the therapeutic range of a drug is known and the other approach when the therapeutic range of the drug is unknown. For kratom, the concentration–response relationship is unknown, so the second approach must be used to interpret the data. In this approach, the 90% confidence interval must fall outside of the 80–125% range to be considered clinically significant [38]. This approach was used to analyze and make conclusions regarding the pharmacokinetic data obtained.

## 3. Results

### 3.1. Formulation Analysis

The commercial products used in this study were analyzed to verify content. The cannabidiol isolate product was found to contain 32.0 mg/mL (>95% label claim) cannabidiol. The OPMS Gold product contained 11.8 mg/mL mitragynine, 2.8 mg/mL speciociliatine, 2.2 mg/mL paynantheine, and 1.5 mg/mL speciogynine. These alkaloid ratios are consistent with other commercially available products as well as dried leaf kratom material, and as such, are representative of real-world kratom use [34]. Based on these results, the doses used were 1.5 mL/kg and 0.8 mL/kg for cannabidiol isolate and kratom extract, respectively. 

### 3.2. Single-Dose Pharmacokinetic Study

**Figure 2:**
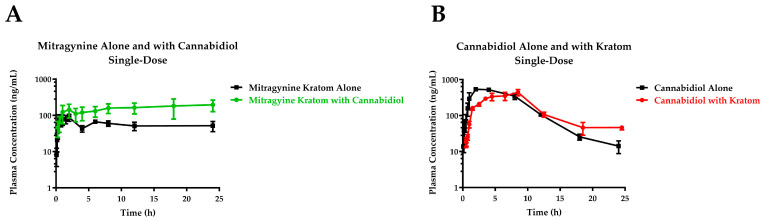
Mitragynine (**A**) and cannabidiol (**B**) plasma concentration–time curves following a single oral dose of kratom product (OPMS 0.8 mL/kg; mitragynine equivalent dose of 9.4 mg/kg) and cannabidiol (50 mg/kg) alone or in combination in male rats. Values represent the mean ± the standard error of the mean.

With cannabidiol coadministration, the maximum plasma concentration (C_max_) of mitragynine increased 2.3-fold. The systemic exposure over the time interval 0–24 h (AUC_0–24_) increased 2.8-fold (Table 1). The 90% confidence interval for both the maximum concentration and exposure increase was well above 125%, indicating that the increase in exposure may be clinically significant. There was also an increase in the time to reach maximum concentration (T_max_) and a delay in the absorption of mitragynine (Figure 2A). 

There was an increase in the T_max_ for cannabidiol upon coadministration with kratom, though more moderate than the change in T_max_ for mitragynine, as well as a slight decrease in the C_max_ (Figure 2B). But the overall exposure, AUC_0–24_, was similar, and the range of 90% confidence interval values fell within 0.8–1.1 (Table 2). 

Minor kratom alkaloids were also quantified after a single oral dose of cannabidiol and kratom. All minor alkaloids showed an overall increase in the C_max_, T_max_, and AUC_0–24_, similar to mitragynine (Table 3). The exposure fold increase for minor alkaloids was 4- to 6-fold. There was also an increase in the concentration of the active metabolite, 7-hydroxymitragynine, but the metabolite to parent (mitragynine) exposure ratio percent remained similar (3.1 and 3.5% without and with cannabidiol, respectively).

### 3.3. Multiple-Dose Pharmacokinetic Study

**Figure 3:**
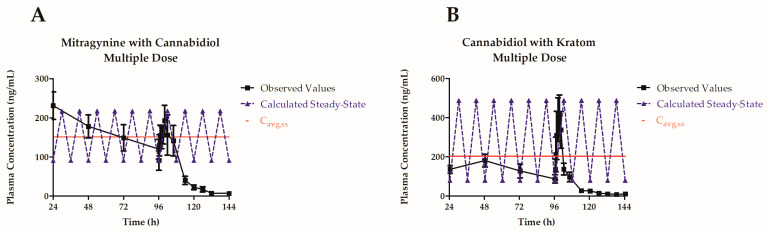
Mitragynine (**A**) and cannabidiol (**B**) plasma concentration–time curves following multiple oral doses of kratom product (OPMS 0.4 mL/kg; mitragynine equivalent dose of 4.7 mg/kg) and cannabidiol (25 mg/kg). Values represent the mean ± SEM. Dashed lines represent the calculated steady-state plasma concentration–time curves for each compound.

The single-dose pharmacokinetic study results demonstrated that with coadministration of cannabidiol, a potential clinically relevant increase in the exposure of kratom alkaloids occurs. To further explore these changes, a steady-state pharmacokinetic study of concomitantly dosed cannabidiol and kratom product was performed (Figure 3) to see if any time-dependent inhibition and/or induction of metabolic enzymes or other pharmacokinetic processes occurred once the compounds reached steady-state.

The exposure of mitragynine over the dosing interval (AUC/τ) was 1813.1 ± 416.6 h·ng/mL, and the steady-state concentration (C_avg,ss_) of mitragynine was 151.1 ± 34.7 ng/mL while the AUC/τ of cannabidiol was 2446.0 ± 615.1 h·ng/mL, and the C_avg,ss_ was 203.8 ± 51.3 ng/mL (Table 4). Neither compound showed a strong potential for accumulation, as both had accumulation ratios of less than 2.0. The fluctuation for mitragynine was close to 100%, which indicates the dosing interval was appropriate. Cannabidiol had a larger fluctuation, so in future studies the dose or dosing interval of cannabidiol could be modified to reduce the fluctuation.

The steady-state plasma concentration–time profiles for mitragynine and cannabidiol were graphed using the non-compartmental analysis data and plotted with the observed data (Figure 3). 

The single-dose and multiple-dose pharmacokinetic parameters were also compared. The pharmacokinetic properties remained largely unchanged between single- and multiple-dose, which reveals that no time-dependent pharmacokinetic interactions occur for cannabidiol and kratom (Table 5).

## 4. Discussion

Often, there are changes in pharmacokinetic properties of compounds when given together, but these changes are only considered clinically significant when the magnitude of the change is large enough to result in a change in pharmacodynamic outcomes. 

Any product that produces a ≥2-fold change in exposure ratio often comes with dosage warnings and suggestions [39]. Based on this, cannabidiol should be reported as contributing to a significant increase (3- to 6-fold for various alkaloids) in kratom alkaloid exposure. Epidiolex™ has warnings for coadministration with drugs metabolized by certain cytochrome P450 (CYP) enzymes, but these do not include CYP3A4, which is the primary enzyme responsible for kratom alkaloid metabolism [36].

Studies looking at the effect of coadministration of cannabidiol with other drugs have been performed. In a single-dose study examining the pharmacokinetics of clobazam, a benzodiazepine used to decrease seizure frequency in epileptic patients, a dose of 5 mg/kg clobazam and 12 mg/kg cannabidiol was delivered intraperitoneally in mice. Similarly to what was seen when kratom was coadministered with cannabidiol, there was a delay in the absorption of clobazam and an increase in exposure [15]. The effect of multiple-dose administration of cannabidiol on the pharmacokinetics of clobazam, stiripentol, and valproate in humans has also been reported. The behavior of the water-soluble drug valproate was unchanged in the presence of cannabidiol, while the more lipophilic drugs (clobazam and stiripentol) had increases in exposure with cannabidiol coadministration [22]. 

All kratom alkaloids had increased exposure with concomitant cannabidiol administration. The safety and toxicity of minor kratom alkaloids have not been reported, so caution must be exercised if cannabidiol and kratom products are coadministered until more information is known. One alkaloid of concern is speciociliatine, which has a higher affinity for µ-opioid receptors than mitragynine, so it may have abuse liability and could be the reason that some kratom users experience withdrawal symptoms upon abrupt cessation of kratom [29]. Recent pharmacokinetic analysis of whole kratom products in humans indicates that speciociliatine exhibits higher exposure as compared to mitragynine despite a 4-fold lower dose delivered [8]. Another diastereomer of mitragynine, mitraciliatine, also had high exposure after kratom dosing in humans and is a partial µ-opioid receptor agonist [40]. Additionally, corynantheidine, which was present in quantities of less than 1% w/v in the kratom product dosed in this study, had measurable concentrations and appreciable exposure due to its higher oral bioavailability (50%) [41]. The results of this study are concerning, as individuals concurrently using kratom and cannabidiol may have increased exposure of all of the alkaloids found in a complex kratom product, most of which have little to no safety data to support their use.

Interestingly, the metabolite to parent (mitragynine) exposure ratio percent of 7-hydroxymitragynine remained similar (3.1 and 3.5% without and with cannabidiol, respectively). As there was an increase in exposure of the parent compound, it was expected that this would be due to a decrease in metabolism, but this was not the case for 7-hydroxymitragynine despite it being primarily metabolized by CYP3A and cannabidiol being a competitive inhibitor of CYP3A [42,43]. Additional metabolites of mitragynine have now been characterized in rats [44], so future analysis should include those metabolites to determine how their formation may be affected by coadministration of cannabidiol. 

Because there is greater exposure of kratom alkaloids over the dosing interval, chronic dosing of cannabidiol and kratom may lead to increased pharmacodynamic effects or unwanted side effects due to an increase in the exposure of a minor kratom alkaloid with yet unknown pharmacology. 

A major limitation of this study was that it was only performed in male rats. Sex differences have been reported in the pharmacokinetics of cannabidiol [45,46]. In addition, limited clinical data in humans indicated three female participants withdrew from study due to nausea and/or vomiting, which may indicate that females are more sensitive to kratom than males [8,10]. Future studies must investigate female pharmacokinetics to reveal any sex differences. 

## 5. Conclusions

This study examined, for the first time, the potential pharmacokinetic interactions when cannabidiol and kratom are coadministered. These products are commercially available, and survey data suggests that there are individuals in the US taking kratom with cannabidiol. The results demonstrated that when kratom is dosed concomitantly with cannabidiol, the systemic exposure of the psychoactive compounds found in kratom is increased. Caution must be exercised when administering cannabidiol and kratom products because the clinical relevance of the interactions described here is unknown. Further studies to better understand the mechanism of the pharmacokinetic interaction as well as its pharmacodynamic significance are warranted. 

## Figures and Tables

**Table 1:** Pharmacokinetic parameters of mitragynine following a single oral dose of kratom product (9.6 mg/kg mitragynine) or a single concomitant dose of cannabidiol product (50 mg/kg) and kratom product (9.4 mg/kg mitragynine) in male rats. Values represent the mean ± SEM or the mean (90% confidence interval).

Parameter	Kratom Product Alone (*n* = 4) [35]	Kratom Product Plus Cannabidiol (*n* = 5)	Fold Change (90% CI Ratio)
C_max_ (ng/mL)	111.9 ± 15.6	253.4 ± 68.3	2.3 (1.6, 2.7)
T_max_ (h)	3.1 ± 1.7	17.0 ± 4.6	5.4 (24.3, 4.2)
AUC_0–24_ (h·ng/mL)	1306.8 ± 126.1	5354.9 ± 1145.5	2.8 (1.6, 3.7)

C_max_ = maximum plasma concentration; T_max_ = time to reach C_max_; AUC_0–24_ = area under the concentration–time curve from time 0 to 24 h (exposure); CI = 90% confidence interval; SEM = standard error of the mean.

**Table 2:** Pharmacokinetic parameters of cannabidiol following a single oral dose of cannabidiol product (50 mg/kg) or a single concomitant dose of cannabidiol product (50 mg/kg) and kratom product (9.4 mg/kg mitragynine) in male rats. Values represent the mean ± SEM or the mean (90% confidence interval).

Parameter	Cannabidiol Product Alone (*n* = 4)	Cannabidiol Plus Kratom Product (*n* = 5)	Fold Change (90% CI Ratio)
C_max_ (ng/mL)	588.8 ± 38.3	490.9 ± 69.2	0.8 (0.7, 0.9)
T_max_ (h)	3.8 ± 1.5	6.3 ± 1.0	1.7 (3.8, 1.3)
AUC_0–24_ (h·ng/mL)	4400.6 ± 319.2	4182.6 ± 614.5	1.0 (0.8, 1.1)

C_max_ = maximum plasma concentration; T_max_ = time to reach C_max_; AUC_0–24_ = area under the concentration–time curve from time 0 to 24 h (exposure); CI = 90% confidence interval; SEM = standard error of the mean.

**Table 3:** Pharmacokinetic parameters of minor kratom alkaloids and kratom metabolites following a single oral dose of kratom product [35] or a single concomitant dose of cannabidiol and kratom product. Values represent the mean ± the standard error of the mean.

	Kratom Product Alone (*n* = 4) [35]	Kratom Product Plus Cannabidiol (*n* = 5)
Corynantheidine (0.2 mg/kg)		
C_max_ (ng/mL)	3.1 ± 0.5	12.9 ± 2.0
T_max_ (h)	3.1 ± 1.7	12.6 ± 11.4
AUC_0–24_ (h·ng/mL)	30.4 ± 9.1	195.9 ± 111.1
Speciociliatine (2.2 mg/kg)		
C_max_ (ng/mL)	23.8 ± 1.4	68.1 ± 5.9
T_max_ (h)	3.2 ± 1.6	17.0 ± 5.9
AUC_0–24_ (h·ng/mL)	222.7 ± 22.2	989.7 ± 93.7
Paynantheine (1.8 mg/kg)		
C_max_ (ng/mL)	3.0 ± 1.7	19.8 ± 5.8
T_max_ (h)	0.08 ± 0.0	16.2 ± 4.9
AUC_0–24_ (h·ng/mL)	-	290.0 ± 96.4
7-hydroxymitragynine (<0.1 mg/kg)		
C_max_ (ng/mL)	4.0 ± 0.6	17.5 ± 2.3
T_max_ (h)	3.1 ± 1.7	15.0 ± 5.5
AUC_0–24_ (h·ng/mL)	41.0 ± 7.6	189.2 ± 27.7

C_max_ = maximum plasma concentration; T_max_ = time to reach C_max_; AUC_0–24_ = area under the concentration–time curve from time 0 to 24 h (exposure).

**Table 4:** Multiple-dose pharmacokinetic parameters of mitragynine and cannabidiol following a 5-day q12 h dose of 25 mg/kg cannabidiol and 0.4 mg/mL kratom product (Eq. 4.7 mg/kg, mitragynine) in male rats. Values represent the mean ± SEM.

Parameter	Mitragynine	Cannabidiol
C_min,ss_ (ng/mL)	91.1 ± 24.9	80.9 ± 23.7
C_avg,ss_ (ng/mL)	151.1 ± 34.7	203.8 ± 51.3
C_max,ss_ (ng/mL)	217.5 ± 40.0	488.6 ± 96.2
Fluctuation (%)	90.0 ± 11.3	219.0 ± 42.7
Accumulation Index	1.4 ± 0.1	1.8 ± 0.0
AUC_0–∞_ (h·ng/mL)	2908.0 ± 735.0	3519.3 ± 851.1
AUC/τ (h·ng/mL)	1813.1 ± 416.6	2446.0 ± 615.1

C_min,ss_ = minimum plasma concentration at steady-state; C_avg,ss_ = average plasma concentration at steady-state; C_max,ss_ = maximum plasma concentration at steady-state; AUC_0–∞_ = area under the concentration–time curve from time 0 (96 h) extrapolated to infinity (exposure); AUC/τ = area under the concentration–time curve over the dosing interval (12 h); SEM = standard error of the mean.

**Table 5:** Single- versus multiple-dose pharmacokinetic parameters for mitragynine and cannabidiol. Values represent the mean ± SEM.

Parameter	Single-Dose	Multiple-Dose
	Mitragynine	
AUC_0–∞/_dose (h·ng/mL/mg/kg)	578.6 ± 187.9	605.8 ± 153.1
V/F (L/kg)	25.8 ± 7.5	28.7 ± 3.7
CL/F (L/h/kg)	2.3 ± 0.5	3.1 ± 0.5
	Cannabidiol	
AUC_0–∞/_dose (h·ng/mL/mg/kg)	97.8 ± 18.5	140.8 ± 34.0
V/F (L/kg)	164.5 ± 26.3	192.3 ± 42.1
CL/F (L/h/kg)	11.5 ± 1.6	13.0 ± 2.8

AUC_0–∞_/dose = dose-normalized area under the plasma concentration–time curve; CL/F = apparent oral clearance; V/F = apparent volume of distribution; F = bioavailability fraction; SEM = standard error of the mean.

## Data Availability

The data that support findings of this study are available from the corresponding author upon request.
